# Beyond the liver protective efficacy of silymarin; bright renoprotective effect on diabetic kidney disease

**Published:** 2014-07-01

**Authors:** Masoud Amiri, Parisa Motamedi, Leila Vakili, Nahid Dehghani, Fereshte Kiani, Zahra Taheri, Sara Torkamaneh, Parto Nasri, Hamid Nasri

**Affiliations:** ^1^Social Health Determinants Research Center, Shahrekord University of Medical Sciences, Shahrekord, Iran; ^2^Environmental Health Engineering, Engineering Department, Health Faculty, Isfahan University of Medical Sciences, Isfahan, Iran; ^3^Nour Medical Hospital, Isfahan University of Medical Sciences, Isfahan, Iran; ^4^Young Researchers and Elite Club, Isfahan (Khorasgan) Branch, Islamic Azad University, Isfahan, Iran; ^5^Department of Biostatistics and Epidemiology, School of Health, Isfahan University of Medical Sciences, Isfahan, Iran; ^6^Social Health Determinants Research Center, Shahrekord University of Medical Sciences, Shahrekord, Iran; ^7^Department of Physical Education and Sport Sciences, Khorasgan University of Isfahan, Isfahan, Iran; ^8^Department of Nephrology, Isfahan University of Medical Sciences, Isfahan, Iran

**Keywords:** Silymarin, Kidney, Renoprotection, Diabetes mellitus

**Implication for health policy/practice/research/medical education:**

Silymarin possesses antioxidant and antiproteinuric effects in humans and in animal models. Silymarin seem to have two different effects in diabetic patients. Firstly control of blood sugar and secondly, silymarin seems to have the ability to ameliorate the diabetic nephropathy, an effect beyond controlling the blood glucose in diabetics.

Diabetic nephropathy is one of the most important complications of diabetes mellitus (1). Recently much attention has been directed toward the use of silymarin in diabetic nephropathy (2). Silymarin possesses antioxidant and antiproteinuric effects in humans and in animal models (2,3). Silymarin seems to have two different effects in diabetic patients (2,3). Firstly control of blood sugar and secondly, silymarin seems to have the ability to ameliorate the diabetic nephropathy, an effect beyond controlling the blood glucose in diabetics (2-4). In diabetic nephropathy, excessive generation of reactive oxygen species and the overproduction of superoxide, initiate the podocyte injury, which is a major contributor to the pathogenesis of diabetic kidney disease (2-4). Various studies have shown that silymarin should be a good medication to prevent nephropathy-induced premature death in diabetic patients (2-4). Moreover, it was observed that silymarin had a good protective effect against high glucose-induced podocyte injury too (2-5). Accordingly, to find out the kidney protective properties of silymarin and deferoxamine against iron dextran-induced renal iron deposition in male rats, we also conducted an investigation. We studied rats, which were allocated to six groups and injected iron dextran intraperitoneal for a period of 4 weeks every other day, but at the beginning of the third week, they also were subjected to a 2-week (every other day) treatment with vehicle (group 2, positive control), silymarin (group 3), deferoxamine (group 4), silymarin (group 5) or combination of silymarin and deferoxamine (group 6). In this study, the levels of serum creatinine, iron, ferritin, blood urea nitrogen, and nitrite were determined and the kidneys were removed for histopathological investigations. Silymarin and deferoxamine treatments reduced the intensity of the kidney iron deposition, but only in the silymarin group a significant reduction in kidney iron deposition was observed. We concluded that silymarin is a renoprotective herb against injurious insult of iron deposition in the kidneys of animal models (6). Recently, much attention has been made on the possible kidney protective properties of metformin, too (7-9). To find out the potential efficiency of metformin to renal protection against gentamicin-induced acute renal injury and also to examine whether postpone treatment with metformin in acute renal injury, exerts similar benefits on gentamicin-kidney toxicity in rats, we conducted a study on Wistar rats (10). In our study metformin was able to prevent and attenuate gentamicin-induced acute kidney damage (10). Hence, it might be beneficial in patients under treatment with this drug (9-14). Furthermore, silymarin extract could safely be used together with metformin to increase the antioxidant potency and better renoprotection, beyond the control of diabetes (9-14). In addition, Fallahzadeh et al investigated the effect of addition of silymarin to renin angiotensin system inhibitors on proteinuria in type 2 diabetic patients with overt nephropathy. They found that silymarin was able to reduce urinary excretion of albumin, TNF-a, and malondialdehyde in patients with diabetic kidney disease. This might be considered as a novel addition to the anti-diabetic nephropathy (15). Thus, silymarin extract could safely co-administered together with metformin to increase the antioxidant potency and better kidney protection, beyond the control of blood glucose (7-10). Also, according to the kidney protective efficacy of silymarin in our study and its blood glucose regulatory effects by previous investigators and also effective reduction of urinary albumin excretion in the study of Fallahzadeh et al., it is possible that, the combination of metformin, silymarin and a renin-angiotensin system inhibitor or angiotensin receptor blocker may have additive kidney protective property, beyond controlling the blood sugar, to prevent or to slow the progression of diabetic nephropathy (12-15). In this regard, to better understand the renal protective effect of silymarin, especially when combined with metformin, more experimental rat models or clinical studies suggests.

## Authors’ contributions

All authors contributed to the paper equally.

## Conflict of interests

The authors declared no competing interests.

## Ethical considerations

Ethical issues (including plagiarism, misconduct, data fabrication, falsification, double publication or submission, redundancy) have been completely observed by the authors.

## Funding/Support

None.
